# The median effective dose of hyperbaric ropivacaine and lateral decubitus time for unilateral spinal anesthesia in lower extremity varicose vein surgery: a dose- and time-finding study

**DOI:** 10.3389/fphar.2026.1975353

**Published:** 2026-10-21

**Authors:** Jun Xu, Jiaying Zhou, Haibo Chen, Chao Lin, Wenlin Xian, FangJun Wang

**Affiliations:** 1 The Affiliated Hospital of North Sichuan Medical College, Nanchong, China; 2 The Integrated Traditional Chinese and Western Medicine Hospital of Liangshan Yi Autonomous Prefecture, Xichang, China; 3 The People’s Hospital of Yilong County, Nanchong, China

**Keywords:** hyperbaric ropivacaine, lateral decubitus time, lower extremity varicose vein surgery, motor block, unilateral spinal anesthesia

## Abstract

**Background:**

The aim of this study was to determine the median effective dose (ED50) of 0.5% hyperbaric ropivacaine for motor block in the non-surgical extremity and the median effective lateral decubitus time (ET50) for preventing motor block in the non-surgical extremity during lower extremity varicose vein surgery.

**Methods:**

Sixty patients undergoing unilateral lower extremity varicose vein surgery under combined spinal-epidural anesthesia were enrolled in two sequential phases: a dose-finding phase (*n* = 30) and a time-finding phase (*n* = 30). All patients received intrathecal 0.5% hyperbaric ropivacaine. A modified Dixon up-and-down method was applied sequentially. In the dose-finding phase, the lateral decubitus position was maintained for 20 min. In the subsequent time-finding phase, patients received the ED5 of hyperbaric ropivacaine estimated in the dose-finding phase, corresponding to a 5% probability of motor block in the non-surgical extremity. Successful prevention of motor block was defined as a modified Bromage score <1. For probit analysis, motor block (modified Bromage score ≥1) was coded as the positive response in the dose-finding phase, whereas successful prevention of motor block in the non-surgical extremity was coded as the positive response in the time-finding phase. The ED50 and ET50 were treated as the primary estimates, whereas additional quantile estimates derived from the fitted probit models were considered exploratory.

**Results:**

The ED50 of 0.5% hyperbaric ropivacaine for motor block in the non-surgical extremity was 12.683 mg (95% CI: 12.367-12.997 mg). The ET50 of lateral decubitus time for preventing motor block in the non-surgical extremity was 13.887 min (95% CI: 13.390-14.338 min). The exploratory ED5, corresponding to a 5% probability of motor block, was 11.956 mg (95% CI, 10.304-12.303 mg), while the exploratory ET95 was 14.957 min (95% CI, 14.455-17.016 min). Adverse events were infrequent, and intraoperative hemodynamics remained stable.

**Conclusion:**

The ED50 of 0.5% hyperbaric ropivacaine for non-surgical extremity was 12.683 mg (95%CI: 12.367-12.997 mg), and the ET50 of lateral decubitus time for preventing motor block was 13.887 min (95% CI: 13.390-14.338 min).

**Clinical Trial Registration:**

[www.chictr.org.cn/showproj.html?proj=239741], identifier [ChiCTR2400088340].

## Introduction

Compared to general anesthesia, spinal anesthesia preserves respiratory function, shortens postoperative recovery time, reduces perioperative opioid consumption, and significantly shortens hospital stays (Neuman et al., 2014; Pierce et al., 2017; Capdevila et al., 2020; Stewart et al., 2020; Turner et al., 2021). However, spinal anesthesia can result in motor block in both the surgical and the non-surgical lower extremities, elevated intraoperative hemodynamic variability, delayed recovery of motor function, and a higher incidence of urinary retention (Casati et al., 1998; Fanelli et al., 2000). In contrast, unilateral spinal anesthesia limits the motor block to the surgical extremity, maintains stable intraoperative hemodynamics, promotes rapid recovery of motor function, and reduces the incidence of urinary retention. Furthermore, the motor function of the non-surgical lower extremity is preserved during unilateral spinal anesthesia, reducing the physical workload of medical staff during the perioperative period (Casati and Fanelli, 2001; Moosavi Tekye and Alipour, 2014). These advantages are particularly relevant to ambulatory and short-stay lower-extremity surgery, as rapid motor recovery and early readiness for discharge are key components of enhanced postoperative recovery (Tazreean et al., 2022).

In unilateral lower extremity varicose vein surgery, the surgical procedure is only performed on one lower limb. Therefore, unilateral spinal anesthesia maybe a better anesthesia strategy for unilateral lower extremity varicose vein surgery (Iyyankutty and Chitra, 2024). Unilateral spinal anesthesia with a hyperbaric local anesthetic relies primarily on the density difference between the injectate and cerebrospinal fluid, with gravity governing its spread within the subarachnoid space (Casati et al., 1998). In the present study, 0.5% hyperbaric ropivacaine was selected because it provides reliable spinal anesthesia with a less intense and shorter duration of motor block than bupivacaine, characteristics that may favor early motor recovery in ambulatory lower extremity surgery (Whiteside et al., 2003). In addition, the 0.5% concentration has been used in previous clinical studies and was considered sufficient to provide surgical side anesthesia while allowing adjustment of the total intrathecal dose (Wang et al., 2022).

The patient position, dosage of local anesthetic, and lateral decubitus time are crucial for unilateral spinal anesthesia (Casati et al., 1998; Al Malyan et al., 2006). Patients should be placed in the lateral decubitus position with the surgical side facing down and maintained in this position until the local anesthetic is adequately fixed to the neural tissue, allowing gravity to promote preferential deposition of the hyperbaric solution around the surgical side nerve roots and thereby producing sensory and motor block confined to the surgical side. An excessive dose of ropivacaine for unilateral spinal anesthesia may result in bilateral block, whereas an insufficient dose may lead to inadequate duration of block, failing to meet the surgical requirements (Wang et al., 2022). Meanwhile, an insufficient duration may prevent adequate fixation of the local anesthetic to the neural structures on the surgical side, allowing contralateral spread after repositioning, and an excessively prolonged duration may unnecessarily extend anesthetic preparation and compromise workflow efficiency (Danelli et al., 2002; Casati et al., 2004). However, the optimal dose of 0.5% hyperbaric ropivacaine and duration of lateral decubitus position for unilateral spinal anesthesia remain unclear.

Therefore, the aim of this study was to determine the ED50 of 0.5% hyperbaric ropivacaine associated with a 50% probability of motor block in the non-surgical extremity during unilateral spinal anesthesia. Based on the dose-finding phase, the ED5 of hyperbaric ropivacaine, corresponding to a 5% probability of motor block in the non-surgical extremity, was then used as the fixed intrathecal dose to determine the ET50 of lateral decubitus time for preventing motor block. These findings may provide reference estimates for further investigation of ropivacaine dose and lateral positioning strategies during unilateral spinal anesthesia.

## Materials and methods

### Study design

The ethics committee of the Affiliated Hospital of North Sichuan Medical College (2024ER424-1) approved this study, and written informed consent was obtained from all patients enrolled in the trial. The study was registered at the Chinese Clinical Trial Registry (www.chictr.org.cn/showproj.html?proj=239741, ChiCTR2400088340; principal investigator: Jun Xu; Date of registration: 15 August 2024). The ethical approval process complies with the requirements of Good Clinical Practice, the Declaration of Helsinki, and relevant Chinese laws and regulations.

Patients scheduled for elective unilateral lower extremity varicose vein surgery at the Affiliated Hospital of North Sichuan Medical College between 15 August 2024 and 1 November 2024, aged 18–65 years, with ASA physical status I-III and a body mass index (BMI) of 18.5–23.9 kg·m^-2^, were prospectively enrolled. The exclusion criteria were spinal or nerve root disease, spinal deformity or trauma, severe back pain, infection at the puncture site, coagulopathy, raised intracranial pressure, known allergy to lidocaine or ropivacaine, participated in other drug trials within 3 months, motor abnormality, auditory impairment, dementia, and psychiatric illness precluding cooperation. The withdrawn criteria were unable to tolerate the lateral decubitus position, the duration of surgery exceeded 2 h, spinal puncture failed, epidural rescue anesthesia or conversion to general anesthesia.

### Scoring criteria and blinding

A modified Bromage score was used to assess motor block in both lower extremities: 0 = no motor block; 1 = inability to lift the leg; 2 = inability to bend the knee; 3 = inability to bend the ankle (Lin et al., 2025). Motor block in both lower extremities was assessed every minute during the first 15 min after subarachnoid injection of ropivacaine and then every 10 min until the modified Bromage score of the surgical lower extremity returned to 0. The sensory block level was determined using the pinprick test (Luck et al., 2008).

The combined spinal-epidural procedure was performed by an anesthesiologist who was aware of the assigned drug dose and lateral positioning duration. This anesthesiologist was not involved in outcome assessment. Motor block assessment was performed by an independent investigator who did not participate in the anesthesia procedure and was blinded to both the administered drug dose and the duration of lateral positioning. The patients, surgeons, operating room nurses, and ward nurses were blinded to the grouping. Intraoperative management followed a standardized protocol with predefined criteria for hemodynamic intervention.

### Preparation of 0.5% hyperbaric ropivacaine

Hyperbaric ropivacaine was prepared immediately before intrathecal injection by mixing 10 mL of 1% ropivacaine with 10 mL of 10% glucose, yielding 20 mL of 0.5% ropivacaine with 5% glucose. The same preparation method was used throughout the study to ensure consistency of the intrathecal solution across all patients.

### Anesthetic procedure

All patients were fasted for 8 h and abstained from drinking for 2 h preoperatively without preoperative medication. After arriving at the operating room, standard monitoring was instituted, including electrocardiography (ECG), systolic, diastolic, and mean arterial pressures (SBP, DBP, MAP), heart rate (HR), and peripheral oxygen saturation (SpO_2_). Supplemental oxygen was delivered via face mask at 6 L·min^-1^, and compound sodium chloride solution was administered intravenously at 8–10 mL·kg^-1^·h^-1^ (Wang et al., 2022).

Before unilateral spinal anesthesia was performed, the operating table was tilted 30° to the Trendelenburg position. Combined spinal-epidural anesthesia was performed with the patient in the lateral decubitus position with non-surgical side uppermost. After ultrasound-guided identification of the L_3-4_ interspace and sterile preparation, 2% lidocaine was infiltrated locally. An epidural puncture was performed using the loss-of-resistance technique, and a spinal needle was then advanced through the epidural needle to puncture the dura and arachnoid mater. After free cerebrospinal fluid (CSF) outflow without blood aspiration, the needle orifice was directed towards the surgical side, and 0.5% hyperbaric ropivacaine was injected intrathecally at 0.1 mL·s^-1^. Following the removal of the spinal needle, an epidural catheter was inserted 3–5 cm into the epidural space. The indwelling epidural catheter was reserved exclusively for intraoperative rescue analgesia. Patients who required epidural rescue were considered to have suffered from spinal anesthesia failure, were withdrawn from the study protocol, and were excluded from the dose-response analysis. For rescue management, after negative aspiration for blood or cerebrospinal fluid, a test dose of 3 mL of 1.5% lidocaine with epinephrine 1:200,000 was administered. If catheter misplacement was not suspected, 2% lidocaine was administered epidurally in 3–5 mL increments every 3–5 min until adequate surgical anesthesia was achieved (Plaat et al., 2022; Girard and Savoldelli, 2024). Sensory block level was assessed by pinprick, and once the block level reached T_10_ on the surgical side, the operating table was returned to the horizontal position.

The blood pressure was maintained within ±20% of baseline values. Hypotension was defined as a decrease in blood pressure > 20% from baseline or a sustained SBP < 90 mmHg, and treated with intravenous injection of 6 mg ephedrine. Bradycardia was defined as a HR < 50 beats·min^-1^ with a downward trend, and treated with intravenous injection of 0.5 mg atropine.

### Up-and-down sequential design

Dose-finding phase: the lateral decubitus position was maintained for 20 min (Wang et al., 2022), and the initial dose of 0.5% hyperbaric ropivacaine was 12 mg (Cantürk et al., 2012). For each subsequent patient, the dose was adjusted in 0.5 mg increments according to the modified Bromage score of the non-surgical extremity in the preceding patient. A score of ≥1, indicating motor block in the non-surgical extremity, prompted a 0.5 mg decrease in the dose for the next patient, whereas a score of <1, indicating absence of motor block, prompted a 0.5 mg increase.

Time-finding phase: Based on the dose-finding phase, the ED5 of 0.5% hyperbaric ropivacaine was estimated and used as the fixed intrathecal dose for all patients. The initial lateral decubitus position time was 15 min (Wang et al., 2022), measured from completion of the intrathecal injection. For each subsequent patient, the lateral decubitus time was adjusted in 1min increments according to the modified Bromage score of the non-surgical extremity in the preceding patient after repositioning to the supine position. A score of ≥1, indicating motor block, prompted a 1min increase in lateral decubitus time for the next patient, whereas a score of <1, indicating absence of motor block, prompted a 1min decrease.

### Measurements

The primary outcomes were the ED50 of hyperbaric ropivacaine for motor block in the non-surgical extremity and the ET50 of lateral decubitus time for preventing motor block in the non-surgical extremity. Secondary outcomes included the ED5 of hyperbaric ropivacaine, the ET95 of lateral decubitus time, and the onset time and duration of sensory and motor block. Sensory block onset time was defined as the time from the intrathecal injection of ropivacaine to loss of pinprick sensation at the T_10_ dermatome, and sensory block duration as the time from the intrathecal injection of ropivacaine to regression to the L_1_ dermatome. The motor block onset time was defined as the time from the intrathecal injection of ropivacaine to a Bromage score of 3 in the surgical lower extremity, and the motor block duration as the time from injection of ropivacaine to regression to a Bromage score of 1 in the surgical lower extremity. MAP and HR were recorded at 5 min before anesthesia (T_0_), attainment of the T_10_ sensory block (T_1_), 15min (T_2_), 30min (T_3_), and 45 min (T_4_) after intrathecal injection of ropivacaine, and immediately after surgery (T_5_). Adverse events, including hypotension, bradycardia, urinary retention, nausea and vomiting, shivering, respiratory depression, and headache, were also documented.

### Statistical analysis

According to a previous study, a sample size of 20–40 patients would provide reliable estimates of the target dose (Pace and Stylianou, 2007). Thus the sample size was set at 30 patients in the dose group and 30 patients in the time group, with a total of 60 patients.

The data were analysed by using SPSS version 29.0 for Windows (IBM Corp, Armonk, NY, USA). The normality of distribution of continuous variables was tested using the Shapiro-Wilk test. Continuous variables with normal distribution were presented as mean ± SD, whereas non-normal continuous variables were presented as median (interquartile range). Categorical variables were presented as counts (percentages). Data measured at multiple time points within each group were analysed using one-way repeated-measures analysis of variance. Sphericity was tested with Mauchly’s test, and when violated, the Greenhouse-Geisser correction was applied. Post-hoc analysis was performed using the SNK *post hoc* test to detect pairwise differences with Bonferroni correction.

Probit regression was used to characterize the dose-response and time-response relationships. For the dose-response analysis, motor block in the non-surgical extremity (modified Bromage score ≥1) was coded as the positive response and absence of motor block (modified Bromage score <1) as the negative response, such that the probability of motor block in the non-surgical extremity increased with increasing ropivacaine dose. For the time-response analysis, successful prevention of motor block (modified Bromage score <1) was coded as the positive response, such that the probability of preventing motor block increased with increasing lateral decubitus time. The ED50 and ET50 with their 95% confidence intervals were treated as the primary estimates, whereas the ED5 and ET95 derived from the fitted probit models were considered exploratory estimates. Model slopes with 95% confidence intervals were also estimated. All statistical tests were two-sided, and *P* < 0.05 was considered statistically significant. For multiple comparisons, the significance level for pairwise comparisons was adjusted to 0.05 divided by the number of comparisons.

## Results

A total of 66 patients assessed for eligibility between August 15 and 1 November 2024, 34 patients were assessed in the dose-finding phase and 32 patients in the time-finding phase. In the dose-finding phase, one patient declined participation, 33 patients were enrolled, and three patients were subsequently withdrawn because of spinal anesthesia failure (*n* = 1) or surgical duration >2 h (*n* = 2), with 30 patients included in the analysis. In the time-finding phase, one patient declined participation, 31 patients were enrolled, and one patient was subsequently withdrawn because of surgical duration >2 h, with 30 patients included in the analysis (Figure 1). Demographic data were shown in Table 1.

**FIGURE 1:**
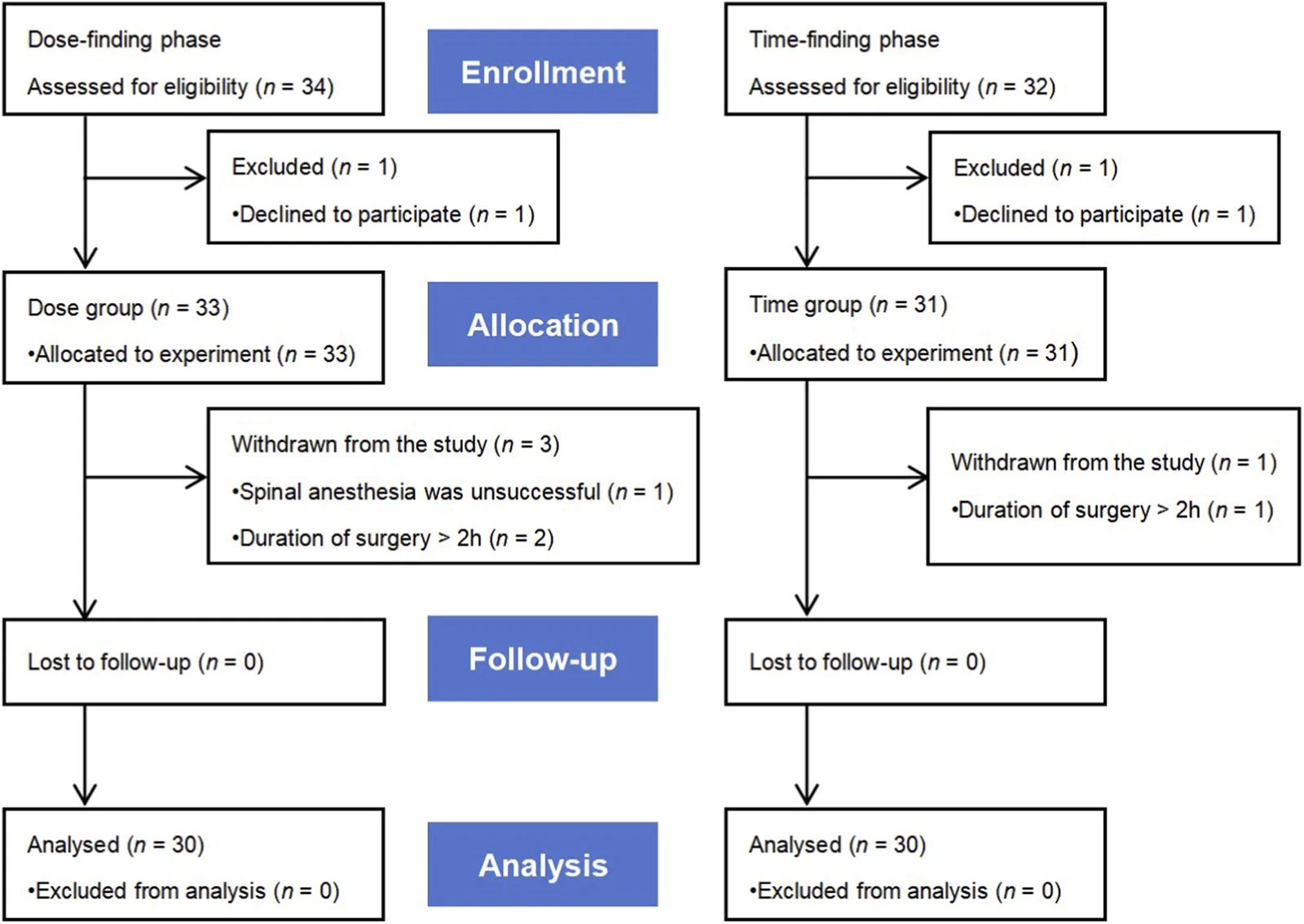
Consort flow diagram.

**TABLE 1:** Demographic data.

​	Dose group (*n* = 30)	Time group (*n* = 30)
Age, mean ± SD (yr)	60.3 ± 4.3	57.9 ± 6.7
Weight, mean ± SD (kg)	65.8 ± 6.7	65.2 ± 7.1
Height, mean ± SD (cm)	168.2 ± 7.4	167.3 ± 7.5
Surgery time, mean ± SD (min)	81.6 ± 19.7	81.2 ± 19.1
Blood loss, mean ± SD (mL)	59.0 ± 16	57.0 ± 16.3
Gender, *n* (%)		
Male	17 (56.7)	16 (53.3)
Female	13 (43.3)	14 (46.7)
ASA class, *n* (%)		
I	10 (33.3)	12 (40.0)
II	16 (53.3)	15 (50.0)
III	4 (13.4)	3 (10.0)

Data are presented as mean ± SD or *n* (%). ASA: American society of anesthesiologists.

Probit regression analysis showed that the probability of motor block in the non-surgical extremity increased with increasing ropivacaine dose, with a slope of 2.264 (95% CI, 0.707-3.821; *P* = 0.004). The ED50 of 0.5% hyperbaric ropivacaine, corresponding to a 50% probability of motor block, was 12.683 mg (95% CI, 12.367-12.997 mg). In the time-response analysis, the probability of preventing motor block in the non-surgical extremity increased with increasing lateral decubitus time, with a slope of 1.536 (95% CI, 0.531-2.541; *P* = 0.003). The ET50 was 13.887 min (95% CI, 13.390-14.338 min). The exploratory ED5, corresponding to a 5% probability of motor block, was 11.956 mg (95% CI, 10.304-12.303 mg), while the exploratory ET95 was 14.957 min (95% CI, 14.455-17.016 min). The sequential allocation diagrams and corresponding dose-response and time-response curves are shown in Figures 2, 3 and Figures 4, 5, respectively.

**FIGURE 2:**
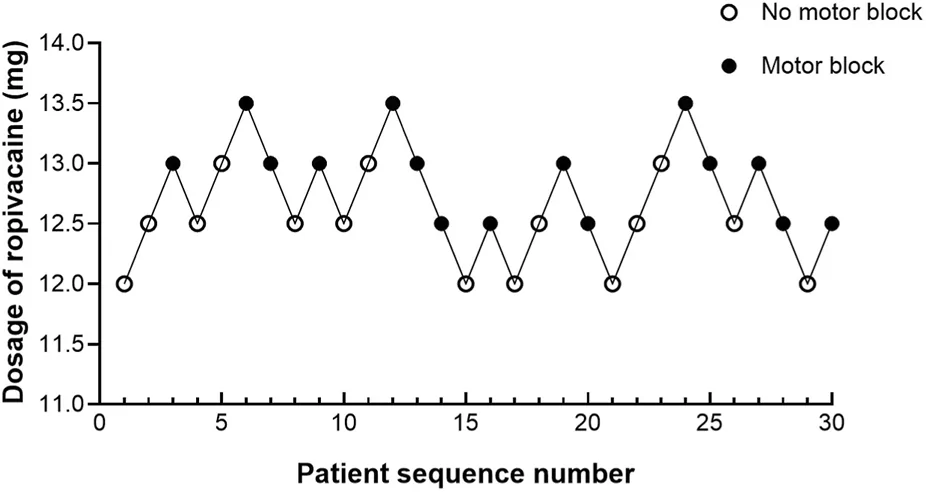
Sequential dose allocation and individual responses to intrathecal ropivacaine in the dose-finding phase. The x-axis represents the patient sequence number, and the y-axis represents the dose of ropivacaine. Solid circles indicate motor block in the non-surgical extremity (modified Bromage score ≥1), whereas blank circles indicate absence of motor block (modified Bromage score <1).

**FIGURE 3:**
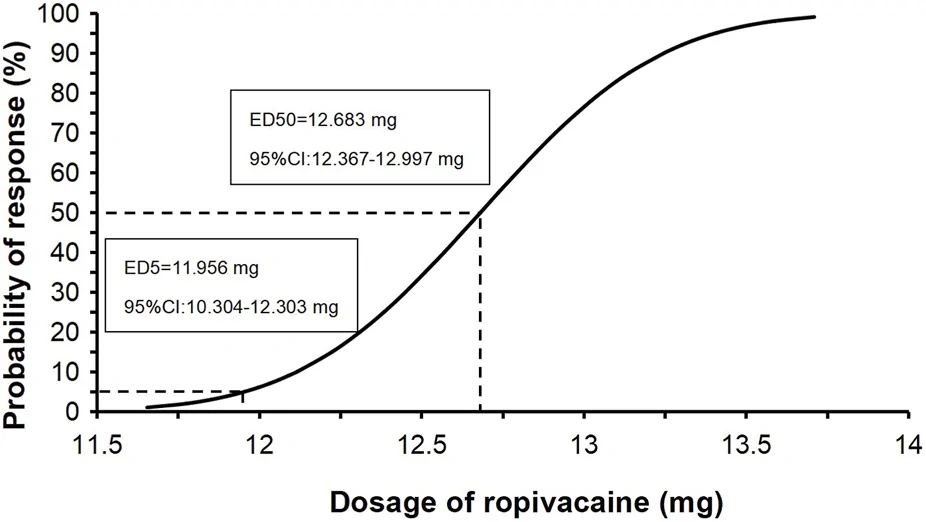
Probit dose-response curve for motor block in the non-surgical extremity during unilateral spinal anesthesia. The x-axis represents the dose of hyperbaric ropivacaine, and the y-axis represents the predicted probability of motor block in the non-surgical extremity (modified Bromage score ≥1). The ED50 and ED5 represent the ropivacaine doses associated with 50% and 5% probabilities of motor block in the non-surgical extremity, respectively.

**FIGURE 4:**
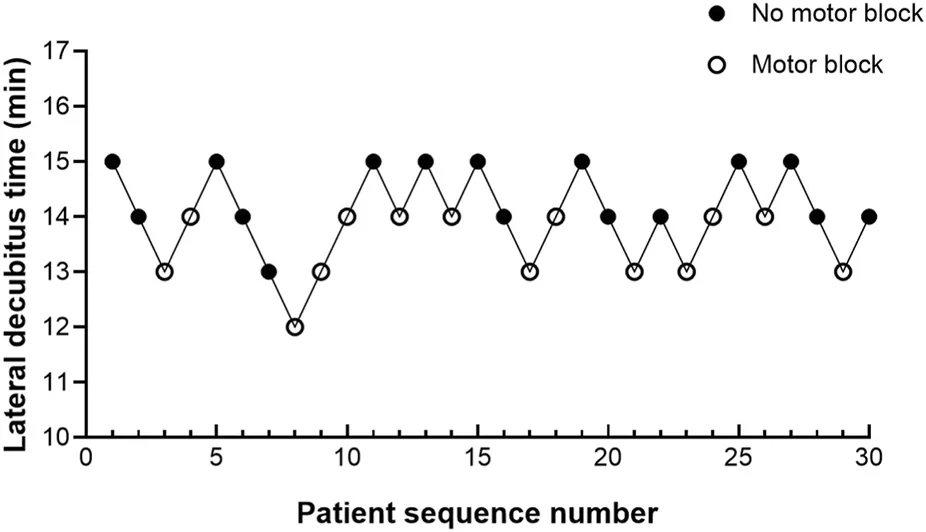
Sequential allocation of lateral decubitus time and individual responses in the time-finding phase. The x-axis represents the patient sequence number, and the y-axis represents the lateral decubitus time. Solid circles indicate absence of motor block in the non-surgical extremity (modified Bromage score <1), whereas blank circles indicate motor block in the non-surgical extremity (modified Bromage score ≥1).

**FIGURE 5:**
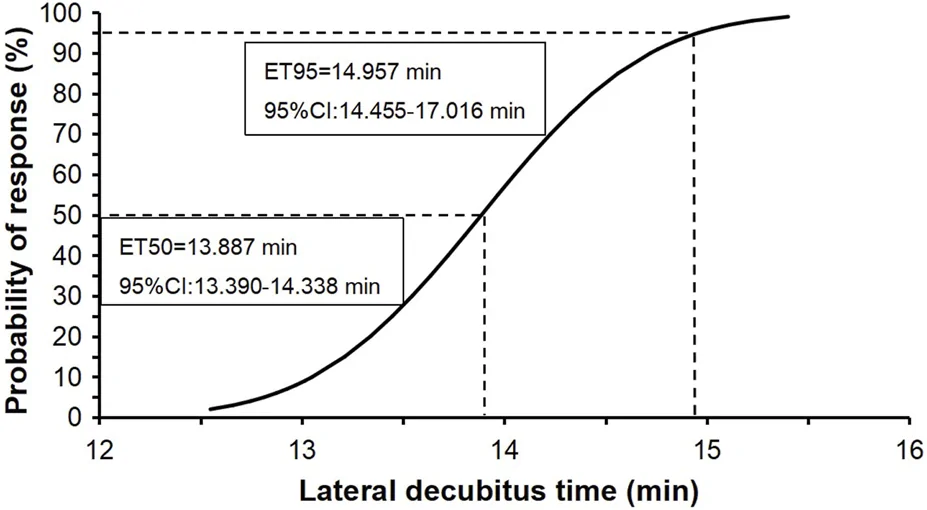
Probit time-response curve for the prevention of motor block in the non-surgical extremity during unilateral spinal anesthesia. The x-axis represents the lateral decubitus time, and the y-axis represents the predicted probability of preventing motor block in the non-surgical extremity (modified Bromage score <1). The ET50 and ET95 represent the lateral decubitus times associated with 50% and 95% probabilities of preventing motor block, respectively.

Characteristics of spinal anesthesia in patients and adverse events were shown in Table 2. The onset time of sensory and motor block were 4.8 ± 0.7, and 7.7 ± 1.0 min in the dose group, and 5.0 ± 0.7, and 7.9 ± 1.0 min in the time group. The duration of sensory and motor block were 151.2 ± 7.0, and 132.0 ± 5.2 min in the dose group, and 150.2 ± 4.3, and 130.3 ± 3.9 min in the time group. In the two groups, no patients suffered from hypotension, nausea and vomiting, shivering, or respiratory depression, and the incidence of bradycardia and urinary retention were 3.3% and 6.7%, respectively. The intraoperative hemodynamics was shown in Table 3. The MAP and HR data violated the assumption of sphericity; therefore, one-way repeated-measures analysis of variance with Greenhouse-Geisser correction was performed. Given the significant time effect, *post hoc* comparisons with Bonferroni correction were performed. Compared with T_0_ (*P* < 0.01 considered significant), the MAP decreased at T_1_-T_5_ both in the dose group and time group (all *P* < 0.001), the HR decreased at T_2_-T_5_ in the dose group, and at T_1_-T_5_ in the time group (all *P* < 0.001).

**TABLE 2:** Characteristics of spinal anesthesia in patients and adverse events.

​	Dose group (*n* = 30)	Time group (*n* = 30)
Onset time of sensory block, mean ± SD (min)	4.8 ± 0.7	5.0 ± 0.7
Onset time of motor block, mean ± SD (min)	7.7 ± 1.0	7.9 ± 1.0
Duration of sensory block, mean ± SD (min)	151.2 ± 7.0	150.2 ± 4.3
Duration of motor block, mean ± SD (min)	132.0 ± 5.2	130.3 ± 3.9
Hypotension, *n* (%)	0 (0)	0 (0)
Bradycardia, *n* (%)	1 (3.3)	1 (3.3)
Urinary retention, *n* (%)	2 (6.7)	2 (6.7)
Nausea and vomiting, *n* (%)	0 (0)	0 (0)
Shivering, *n* (%)	0 (0)	0 (0)
Respiratory depression, *n* (%)	0 (0)	0 (0)

Data are presented as mean ± SD or *n* (%).

**TABLE 3:** Intraoperative hemodynamics.

​	Time points	Dose group (*n* = 30)	Time group (*n* = 30)
MAP, mean ± SD (mmHg)	T_0_	106.3 ± 5.4	107.4 ± 7.2
	T_1_	95.9 ± 4.3^a^	95.2 ± 4.5^a^
	T_2_	95.3 ± 4.2^a^	94.5 ± 4.7^a^
	T_3_	97.1 ± 3.3^a^	98.2 ± 5.0^a^
	T_4_	97.0 ± 5.1^a^	96.0 ± 4.8^a^
	T_5_	98.5 ± 3.3^a^	98.2 ± 3.7^a^
	*F* values	57.802	49.342
HR, mean ± SD (beats/minute)	*P* values	<0.001	<0.001
	T_0_	77.5 ± 7.3	75.4 ± 8.5
	T_1_	73.5 ± 8.2	71.7 ± 8.5^a^
	T_2_	71.5 ± 5.8^a^	68.4 ± 7.5^a^
	T_3_	66.9 ± 5.1^a^	64.8 ± 7.3^a^
	T_4_	65.1 ± 4.9^a^	61.6 ± 5.9^a^
	T_5_	70.2 ± 5.7^a^	66.8 ± 4.9^a^
	*F* values	39.398	55.705
	*P* values	<0.001	<0.001

Data are presented as mean ± SD. ^a^ *P* < 0.01 vs. T_0_. MAP: mean arterial pressure; HR: heart rate; T_0_: 5 min before anaesthesia; T_1_: when the sensory block level reached T_10_; T_2_: 15 min after intrathecal injection; T_3_: 30 min after intrathecal injection; T_4_: 45 min after intrathecal injection; T_5_: immediately at the end of surgery.

## Discussion

The ED50 of 0.5% hyperbaric ropivacaine, corresponding to a 50% probability of motor block in the non-surgical extremity, was 12.683 mg (95% CI, 12.367-12.997 mg). The ET50 of lateral decubitus time for preventing motor block in the non-surgical extremity was 13.887 min (95% CI, 13.390-14.338 min). The incidence of adverse events was low, and intraoperative hemodynamics was stable.

In contrast, Wang et al. found that the ED50 for unilateral spinal anesthesia with 0.5% hyperbaric ropivacaine in older patients undergoing hip arthroplasty were 7.036 mg, which were considerably lower than those in our study (Wang et al., 2022). There are several factors that may be responsible for the dose differences. First, Wang et al. assessed motor and sensory block only in the surgical extremity, without evaluating the non-surgical extremity. The dose they reported therefore reflected the intrathecal dose required to achieve a T_10_ sensory level and provide pain-free conditions during the first hour of surgery, rather than the maximum dose of 0.5% hyperbaric ropivacaine for unilateral spinal anesthesia without inducing bilateral motor block. Second, the mean age of patients in their cohort was approximately 80 years, substantially higher than that in our study (around 60 years), indicating that advanced age may contribute to lower intrathecal dose requirements (Chen et al., 2016). Finally, the mean duration of hip arthroplasty in their study was about 80 min, but the reported dose was sufficient only for the first hour of surgery, and a considerable proportion of patients suffered from inadequate intraoperative analgesia (36.84% of the patients were dissatisfied with anesthesia and 26.32% of the patients reported pain at skin incision), necessitating supplementary epidural administration. In contrast, in our study, all surgical procedures were completed under unilateral spinal anesthesia without additional epidural supplementation, suggesting that in unilateral spinal anesthesia, higher intrathecal doses prolong block duration, thereby providing more reliable conditions for surgery (Esmaoglu et al., 1998).

Comparable dose differences have also been reported with hypobaric ropivacaine. Lin et al. found that in older patients undergoing hip arthroplasty under unilateral spinal anesthesia, the ED50 of 0.4% hypobaric ropivacaine for preventing motor block in the non-surgical extremitywere 11.13 mg (Lin et al., 2025). In contrast, another study reported an ED50 of 6.43 mg for 0.5% hypobaric ropivacaine sufficient to provide adequate anesthesia for the same procedure (Wang et al., 2017). This substantial difference is consistent with our findings and suggests that the ED50 required to prevent motor block in the non-surgical extremityare higher than those for conventional unilateral spinal anesthesia. This difference may be explained by the fact that both our study and that by Lin et al. focused on the dose-response relationship for motor block in the non-surgical extremity while maintaining adequate surgical anesthesia and sufficient block duration, rather than solely determining the dose required for successful unilateral spinal anesthesia.

This study further determined that the ET50 of lateral decubitus time required to prevent motor block in the non-surgical extremity with 0.5% hyperbaric ropivacaine was 13.887 min (95% CI: 13.390-14.338 min). The optimal lateral positioning time for unilateral spinal anesthesia remains variable. Cantürk et al. used 10 min of lateral positioning with 11.25 mg hyperbaric ropivacaine, but the proportion of unilateral spinal anesthesia decreased after repositioning to the supine position (Cantürk et al., 2012). In contrast, longer positioning time of 20–30 min has been reported to improve unilateral motor block selectivity, although excessive delay may compromise operating-room efficiency (Kuusniemi et al., 1997). This variability may be explained by differences in dose, baricity, patient position, injection characteristics, and time-dependent redistribution within the cerebrospinal fluid (Hocking and Wildsmith, 2004). For hyperbaric unilateral spinal anesthesia, the density gradient between the injectate and cerebrospinal fluid is particularly important because it determines gravity-dependent spread toward the dependent nerve roots (Casati and Fanelli, 2001). In this study, 0.5% ropivacaine with 5% glucose was prepared by mixing ropivacaine with 10% glucose, which increases solution density and promotes surgical side distribution during lateral positioning. However, this process is not instantaneous; insufficient positioning may permit redistribution toward the non-surgical side after repositioning (McLeod, 2004). It indicated that lateral positioning time is an important determinant of unilateral block distribution, with an ET50 of 13.887 min under the present study conditions. However, the required duration may vary according to anesthetic and procedural factors.

Intrathecal adjuvants may modify local anesthetic dose requirements. For example, intrathecal dexmedetomidine has been shown to reduce the ED50 of hyperbaric ropivacaine (Tang et al., 2020). Future studies should evaluate whether adjuvant-based regimens can reduce ropivacaine dose or shorten positioning time while preserving non-surgical side motor function.

Clinical study conducted by Cantürk et al. reported that 11.25 mg of 0.4% hyperbaric ropivacaine achieved T_10_ sensory level in 10.2 ± 2.7 min (Cantürk et al., 2012). Similarly, Casati et al. found that 12 mg of 0.5% hyperbaric ropivacaine achieved T_10_ sensory block and a Bromage score of 3 motor block in 10 ± 6 min, both longer than our findings (Casati et al., 2004). The differences may be attributed to the patient position and injection rate: Cantürk used the 15° Fowler position with 1 mL·min^-1^, Casati used the horizontal position with 2 mL·min^-1^, whereas our study employed the 30° Trendelenburg position and faster injection (6 mL·min^-1^), likely enhancing cephalad spread and mixing of the anesthetic (Holman et al., 1997; Serpell and Gray, 1997).

In our study, both groups maintained blood pressure and heart rate within clinically manageable ranges (with MAP and HR reductions of <20% from baseline), and no patients suffered from hypotension or respiratory depression during surgery. This is likely due to the initial sensory block level being controlled at T_10_ in our study. Existing evidence suggests that when the block level of spinal cord does not reach above T_4_, sympathetic and respiratory muscle functions are generally well preserved (Lambert, 1989; Griffith, 1992). Moreover, no patients suffered from headache in our study, which may be attributed to the use of the 25G Sprotte needle that reduces puncture-related trauma and cerebrospinal fluid leakage, thereby lowering the incidence of post-dural puncture headache (Lambert, 1989; Griffith, 1992).

Urinary retention occurred in 6.7% of patients in both groups. Spinal anesthesia may impair bladder function through blockade of the sacral parasympathetic pathways, while perioperative fluid administration may further contribute to postoperative urinary retention. Unilateral spinal anesthesia may theoretically limit the extent of sacral autonomic blockade by restricting neuraxial spread predominantly to the surgical side. However, urinary retention is multifactorial, and its occurrence may also be influenced by patient characteristics, surgical factors, perioperative fluid administration, and analgesic use (Cantürk et al., 2012). Future controlled studies are needed to determine whether unilateral spinal anesthesia reduces the risk of urinary retention and to account for relevant perioperative confounding factors.

This study has several limitations. First, the findings apply primarily to patients with a BMI of 18.5–23.9 kg·m^-2^, and the relationship between BMI and dose or positioning requirements was not specifically examined; therefore, BMI-based individualized regimens require further study in broader populations. Second, the conventional Dixon up-and-down design primarily supports estimation of the 50% response level. Therefore, the ED5 and ET95 derived from probit regression were considered exploratory estimates rather than clinically validated thresholds. Future studies targeting a 95% probability of preventing motor block should use an appropriate sequential design, such as a biased-coin design. Third, because the included procedures required limited muscle relaxation (Iyyankutty and Chitra, 2024), the applicability of this regimen to surgeries with greater relaxation requirements remains uncertain. Finally, baseline lower extremity strength and functional recovery outcomes, including time to ambulation, nursing dependence, and fall-related events, were not systematically assessed; thus, the rehabilitation benefits of preserving non-surgical extremity motor function require confirmation in future trials.

In conclusion, the ED50 of 0.5% hyperbaric ropivacaine for non-surgical extremity was 12.683 mg (95% CI: 12.367-12.997 mg), and the ET50 of lateral decubitus time for preventing motor block was 13.887 min (95% CI: 13.390-14.338 min). These findings provide reference estimates for further investigation of dose and positioning strategies for unilateral spinal anesthesia.

## Data Availability

The original contributions presented in the study are included in the article/supplementary material; further inquiries can be directed to the corresponding author.
